# Aromatase inhibitor-induced bone loss increases the progression of estrogen receptor-negative breast cancer in bone and exacerbates muscle weakness *in vivo*

**DOI:** 10.18632/oncotarget.14139

**Published:** 2016-12-25

**Authors:** Laura E. Wright, Ahmed A. Harhash, Wende M. Kozlow, David L. Waning, Jenna N. Regan, Yun She, Sutha K. John, Sreemala Murthy, Maryla Niewolna, Andrew R. Marks, Khalid S. Mohammad, Theresa A. Guise

**Affiliations:** ^1^ Department of Medicine, Division of Endocrinology, Indiana University, Indianapolis, IN, USA; ^2^ Department of Internal Medicine, Division of Endocrinology, University of Virginia, Charlottesville, VA, USA; ^3^ Department of Cellular and Molecular Physiology, The Pennsylvania State University College of Medicine, Hershey, PA, USA; ^4^ Department of Physiology, Columbia University, New York, NY, USA

**Keywords:** breast cancer, bone, metastasis, aromatase inhibitor, skeletal muscle

## Abstract

Aromatase inhibitors (AIs) cause muscle weakness, bone loss, and joint pain in up to half of cancer patients. Preclinical studies have demonstrated that increased osteoclastic bone resorption can impair muscle contractility and prime the bone microenvironment to accelerate metastatic growth. We hypothesized that AI-induced bone loss could increase breast cancer progression in bone and exacerbate muscle weakness associated with bone metastases. Female athymic nude mice underwent ovariectomy (OVX) or sham surgery and were treated with vehicle or AI (letrozole; Let). An OVX-Let group was then further treated with bisphosphonate (zoledronic acid; Zol). At week three, trabecular bone volume was measured and mice were inoculated with MDA-MB-231 cells into the cardiac ventricle and followed for progression of bone metastases. Five weeks after tumor cell inoculation, tumor-induced osteolytic lesion area was increased in OVX-Let mice and reduced in OVX-Let-Zol mice compared to sham-vehicle. Tumor burden in bone was increased in OVX-Let mice relative to sham-vehicle and OVX-Let-Zol mice. At the termination of the study, muscle-specific force of the extensor digitorum longus muscle was reduced in OVX-Let mice compared to sham-vehicle mice, however, the addition of Zol improved muscle function. In summary, AI treatment induced bone loss and skeletal muscle weakness, recapitulating effects observed in cancer patients. Prevention of AI-induced osteoclastic bone resorption using a bisphosphonate attenuated the development of breast cancer bone metastases and improved muscle function in mice. These findings highlight the bone microenvironment as a modulator of tumor growth locally and muscle function systemically.

## INTRODUCTION

Breast cancer is the most commonly diagnosed cancer in women [1] and the majority of breast tumors are hormone-responsive [2]. Adjuvant endocrine therapies that impair the action of estrogen on breast tissue have become an important treatment strategy, reducing the risk of recurrence and death in women with estrogen receptor (ER)-positive disease [3–5]. Aromatase inhibitors, which block the rate-limiting step of estrogen biosynthesis [6], have replaced selective estrogen receptor modulators (SERMs; e.g., tamoxifen) as the standard of care in postmenopausal breast cancer patients due to improved disease-free survival [7, 8]. AI treatment regimens in the adjuvant setting are typically administered for five years and the extension of AI treatment regimens to ten years is under study [9]. Between 25-50% of women treated with AIs report musculoskeletal toxicities, including joint pain, muscle weakness, and fragility, which result in diminished quality of life and poor compliance [10–19]. Relatively little is known about the molecular mechanism(s) of AI-induced arthralgia or muscle dysfunction. However, it is well established that AI treatment results in significant bone loss and increased fracture risk [17–19]. Our laboratory has had a longstanding interest in investigating how bone loss can impact tumor behavior in the bone microenvironment, a question that is of relevance to breast cancer patients undergoing prolonged AI therapy in the absence of a bone-protective intervention.

During a state of excessive bone resorption, matrix-derived growth factors have been shown to increase the growth of metastatic cancer cells in bone as well as stimulate their expression of osteolytic factors, which further perpetuate the destructive cycle of breast cancer in the skeleton [20]. Additionally, osteoclast-derived proteolytic enzymes have been shown to promote angiogenesis, cancer cell invasiveness, and engraftment at metastatic sites [21]. In the case of estrogen deficiency, a strong systemic increase in oxidative stress and inflammatory tone [22] could further perpetuate bone loss and, ultimately, cancer progression. Effects of bone loss resulting from AI-induced depletion of peripheral estrogen levels on the breast cancer bone metastases have not yet been tested. Our first aim was to assess the role of AI therapy-induced bone loss on the progression of disseminated breast cancer cells *in vivo*.

The impact of AI therapy on skeletal muscle function at the cellular and molecular level remains unknown despite clinical reports of muscle fatigue in AI-treated patients [10–13]. Because the bone matrix can be a source of growth factors, including members of the transforming growth factor (TGF)-β superfamily that affect both bone and muscle [23, 24], a state of high bone turnover could cause release of growth factors into circulation where they act on peripheral tissues. Previous studies in our laboratory have demonstrated that bone-derived TGFβ leads to oxidative overload in neighboring skeletal muscle and impaired muscle contractility in mice with osteolytic bone metastases [25]. The second aim of this study was to evaluate the effect of AI therapy and bone loss on skeletal muscle function in mice with bone metastases.

The overarching hypothesis driving this work is that estrogen deprivation therapy results in a high bone turnover state that increases breast cancer bone metastases and potentiates muscle weakness. It is important to note that we selected a triple negative breast cancer cell line (MDA-MB-231) in order to examine microenvironment-specific effects on tumor growth in the absence of direct effects of inhibition of ER signaling. Here we report that AI treatment causes bone loss and skeletal muscle weakness in OVX mice, and that the prevention of osteoclastic bone resorption attenuates the development of ER-negative breast cancer bone metastases and improves muscle function in AI-treated estrogen deprived mice.

## RESULTS

### Aromatase inhibitor treatment reduced serum 17β-estradiol and trabecular bone volume in OVX nude mice prior to tumor inoculation

**Figure 1:**
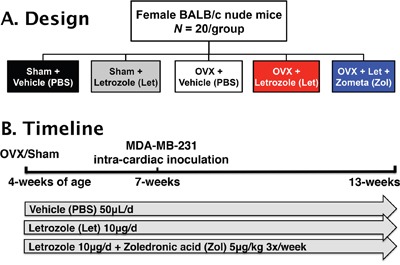
Study design **Panel A.** Female BALB/c athymic nude (n=20/group) mice were randomized into five treatment groups 1) sham + vehicle (PBS), 2) sham + letrozole (Let), 3) ovariectomized (OVX) + vehicle, 4) OVX + letrozole, and 5) OVX + letrozole + zometa (Zol). **Panel B.** At four-weeks of age, mice underwent sham surgery or OVX, and drug treatments commenced 24 hours later. After changes in bone volume and microarchitecture were assessed by micro-computed tomography (μCT) three weeks post-surgery, groups were inoculated in the left cardiac ventricle with 1×105 MDA-MB-231 human breast cancer cells. Mice were followed for six weeks for the development of bone metastases and tissues were collected following euthanasia at 13-weeks of age.

**Figure 2:**
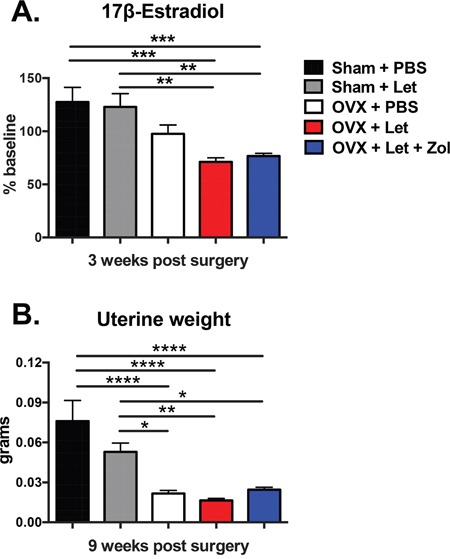
Impact of ovariectomy (OVX) and aromatase inhibitor (letrozole; Let) on circulating 17β-estradiol and on uterine atrophy in nude mice Four-week female athymic nude mice underwent OVX or sham surgery and were treated with vehicle or aromatase inhibitor (letrozole, Let; 10μg/day) ξ00B1;bisphosphonate (zoledronic acid, Zol; 5μg/kg 3x/week; n=20/group). **Panel A.** Serum was collected via retro-orbital puncture at baseline and three weeks after sham/OVX surgery and commencement of drug treatments. Serum 17β-estradiol was measured by immuno-assay (Calbiotech) as per manufacturer protocol, and results are expressed as % of baseline. **Panel B.** At the termination of the study nine weeks post-surgery, uteri were dissected and weighed. Results are expressed as mean ξ00B1;SEM and differences were determined by one-way ANOVA with Tukey's multiple comparisons test where *p<0.05, **p<0.01, ***p<0.001, and ****p<0.0001.

Four-week female athymic nude mice underwent ovariectomy (OVX) or sham surgery and were treated via daily subcutaneous injection with vehicle (PBS, 50μL), or the aromatase inhibitor letrozole (Let, 10μg/d) (Figure 1). A sham-Let group was included as an experimental control in order to assess potential direct effects of letrozole on muscle function in the presence of ovarian estrogen production. A second OVX-Let-treated group was treated with the bisphosphonate zoledronic acid (Zol, 5μ/kg 3x/week) in order to determine the relative importance of bone loss on tumor growth and muscle function in the setting of AI therapy (Figure 1). Prior to the inoculation of tumor cells and three weeks after surgery, serum 17β-estradiol was reduced in OVX-Let and OVX-Let-Zol mice relative to sham groups (Figure 2A). A partial reduction in serum estradiol was observed in OVX-PBS-treated mice relative to sham, although this did not reach statistical significance (Figure 2A). As a terminal and surrogate measure of estrogenic activity [26], uterine weight was recorded at the end of the study (nine weeks post-surgery and five weeks after tumor inoculation). As anticipated, OVX resulted in significant uterine atrophy relative to sham groups regardless of drug treatment, and the addition of aromatase inhibitor to ovary intact mice (sham-Let) resulted in a moderate reduction in uterine weight relative to sham-PBS, though this did not reach statistical significance (Figure 2B).

**Figure 3:**
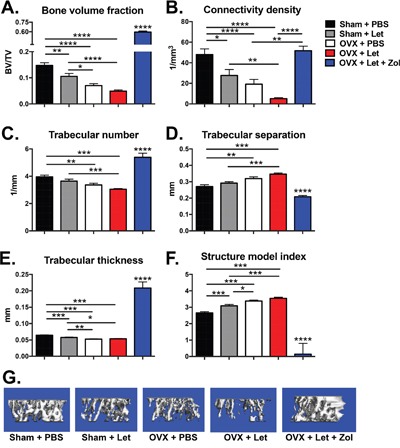
Bone volume and microarchitecture were compromised in nude mice after three weeks of estrogen deprivation treatment Three weeks after OVX/sham surgery and the commencement of drug treatments, mice were anesthetized with isoflurane and bone microarchitecture was assessed in the proximal tibia by micro-computed tomography (μCT40; SCANCO Medical AG). **Panel A.** Trabecular bone volume (BV/TV), **Panel B.** connectivity density (1/mm3), **Panel C.** trabecular number (1/mm), **Panel D.** trabecular separation (mm), **Panel E.** trabecular thickness (mm), and **Panel F.** structure model index are expressed as mean ξ00B1;SEM and differences were determined by one-way ANOVA with Tukey's multiple comparisons test where *p<0.05, **p<0.01, ***p<0.001, and ****p<0.0001. Panel G. Representative reconstructed images of μCT scans showing trabecular bone at the proximal tibia were selected with a BV/TV % most representative of the group mean.

In line with observed estrogenic changes, trabecular bone volume (BV/TV) assessed by bone microcomputed tomography was reduced in sham-Let and OVX-PBS mice (-29% and −52%, respectively) relative to estrogen-replete sham-PBS controls (Figure 3A). The combination of OVX and letrozole (OVX-Let) resulted in 67% reduction in trabecular bone volume relative to sham-PBS mice (Figure 3A). The anti-resorptive zoledronic acid increased trabecular bone volume in the OVX-Let-Zol mice by over three times that of sham-PBS mice and over ten times that of OVX-Let mice after three weeks of treatment (Figure 3A). Bone microarchitectural parameters, including connectivity density, structure model index, and trabecular number, separation and thickness, mirrored changes observed in trabecular bone volume for all treatment groups (Figure 3B-3G). In summary, each microarchitectural property assessed was severely compromised in OVX-Let mice relative to estrogen-replete sham-PBS mice and these maladaptive modifications in trabecular bone were significantly improved by zoledronic acid treatment (Figure 3B-3G).

### Osteolysis and tumor burden were increased in aromatase inhibitor-treated OVX mice

**Figure 4:**
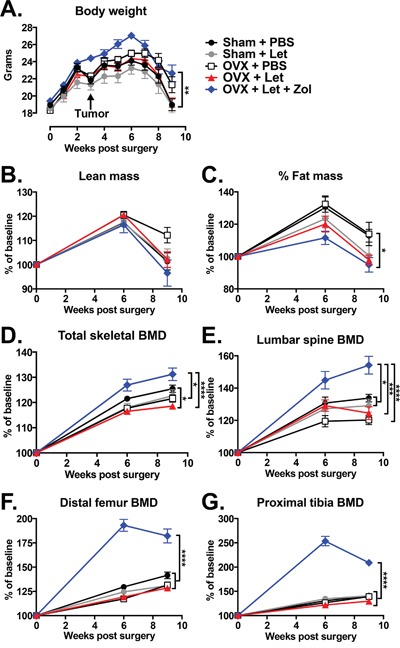
Changes in body composition and bone mineral density (BMD) in estrogen deficient tumor-bearing nude mice Three weeks after the beginning of surgery and drug treatments, groups were inoculated in the left cardiac ventricle with 1×105 MDA-MB-231 human breast cancer cells and followed for six weeks for the development of bone metastases. **Panel A.** Body weight was measured weekly. **Panel B.** Lean mass, **Panel C.** fat mass, **Panel D.** total body BMD, **Panel E.** BMD of the lumbar vertebrae (L4-L6), **Panel F.** distal femur BMD, and **Panel G.** proximal tibia BMD were measured at baseline, six weeks, and nine weeks after the beginning of treatments by dual energy X-ray absorptiometry (DXA). Data are expressed as mean % of baseline ξ00B1;SEM, and differences were determined by two-way ANOVA with Tukey's multiple comparisons test at week nine where *p<0.05, **p<0.01, ***p<0.001, and ****p<0.0001.

Having established an estrogen deficiency-driven high bone turnover state in OVX-Let mice by week three, MDA-MB-231 human breast cancer cells were inoculated into the left cardiac ventricle to generate a model of breast cancer metastatic to bone in order to determine the importance of aromatase inhibitor-induced changes to the bone microenvironment on tumor progression in the skeleton. Weekly body weight measurements were performed as a general assessment of disease progression and overall health [27]. For the duration of the study, OVX-Let-Zol mice maintained a higher average absolute body weight, achieving statistical significance relative to sham-Let and OVX-Let animals (Figure 4A). Body composition analyses by dual energy X-ray absorptiometry (DXA) revealed similar patterns of change in lean mass and fat mass percentage over time between groups, with differences observed only in fat mass percentage at week nine between OVX-Let-Zol and sham-PBS-treated mice (Figure 4B, 4A). The rapid decline in total body weight, lean mass, and fat mass observed in all treatment groups at week six (three weeks post-tumor inoculation) (Figure 4A-4C) is indicative of tumor progression in this model [27].

Changes in bone mineral density (BMD) following the inoculation of tumor cells were assessed by DXA over time. Consistent with the observed increase in trabecular bone volume three weeks after onset of Zol treatment (Figure 3A), BMD was significantly increased in the total skeleton, lumbar vertebrae, distal femur, and proximal tibia in OVX-Let-Zol mice relative to estrogen-replete (sham-PBS), partial estrogen-deprived (sham-Let, OVX-PBS), and total estrogen-deprived mice (OVX-Let) (Figure 4D-4G), confirming that the drug's anti-resorptive effect persisted throughout the study. Importantly, the OVX-Let group had a significant reduction in total skeletal BMD at the nine-week time point relative to sham-PBS (Figure 4D), indicating that AI treatment continued to cause significant bone loss over the course of the nine-week study due to estrogen deficiency and its possible role in cancer-induced osteolysis.

**Figure 5:**
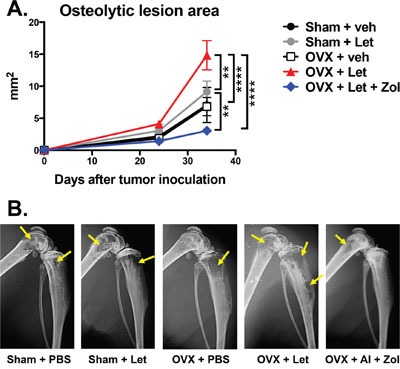
Osteolytic bone metastases assessed by radiography in estrogen deficient nude mice **Panel A.** Osteolytic lesions were measured in anesthetized mice in prone position on d23 and d32 post tumor inoculation using a digital X-ray imager (Kubtec) at 2.7x magnification. Lytic lesion area is reported as total lesion area (mm2) per animal in hind limb and forelimb long bones. **Panel B.** Representative X-rays showing radiolucent lytic lesions (arrows) were selected with lesion areas most representative of the group mean.

**Figure 6:**
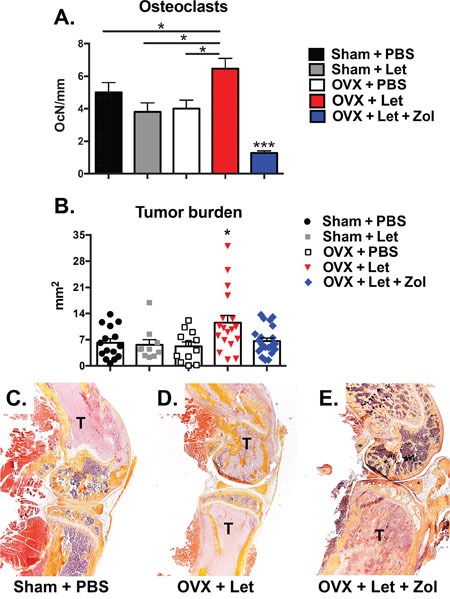
Histological assessment of tumor-bearing bone of estrogen deficient nude mice **Panel A.** Tartrate-resistant acid phosphatase (TRAP)-positive multi-nucleated cells were quantitated at the bone-tumor interface in mid-sagittal sections of the tibia and expressed as number of osteoclasts per mm of interfacing surface (OcN/mm). **Panel B.** Mid-sagittal sections were stained with hematoxylin and eosin (H&E) and total tumor area was measured in the tibia, femur and humerus at 10x magnification. Tumor area is expressed as the combined total tumor area per animal (mm2). **Panel C.** Representative histological images (H&E) showing tumor cells (T) in the distal femur and proximal tibia were selected with tumor areas most representative of the group mean. Differences were determined by one-way ANOVA with Tukey's multiple comparisons test where *p<0.05 and ***p<0.001.

In order to directly assess cancer-induced destruction of bone, radiographs were acquired three and five weeks post-tumor inoculation, and X-rays were carefully examined for radiolucent regions indicative of cancer-mediated osteolysis. Osteolytic lesion area was increased in estrogen deprived OVX-Let mice relative to all treatment groups (Figure 5A, 5B). Lesions were detected in AI-treated OVX mice treated with bisphosphonate (OVX-Let-Zol) (Figure 5A); however, the anti-resorptive effects of Zol drastically reduced the total osteolytic lesion area relative to their estrogen-deprived counterparts (OVX-let) (Figure 5A, 5B). These data were corroborated at the histological level through the quantitation of multinucleated bone-resorbing osteoclasts at the bone-tumor interface in the marrow compartment [27]. Osteoclast numbers were increased in OVX-let mice relative to all groups, and Zol treatment reversed this heightened state of osteoclastogenesis (Figure 6A), in line with the drug's ability to induce osteoclast apoptosis [28].

Having confirmed effects on bone, a primary aim of the study was to evaluate how these AI- and Zol-induced changes to the bone microenvironment could influence tumor progression in the absence of direct inhibition of tumor growth through manipulation of ER signaling. Use of the MDA-MB-231 triple-negative human breast cancer cell line permitted this bone-centric objective. Tumor burden in OVX-Let mice was measured at sites of skeletal metastases in histological sections, and total tumor area (mm^2^) was significantly increased relative to all groups (Figure 6B-6E). Trabecular bone appeared intact in OVX-Let-Zol mice despite the presence of tumor cells (Figure 6E). The overall reduction in tumor area in Zol-treated mice relative to OVX-Let mice (Figure 6D) is likely attributed to the prevention in osteoclastic bone resorption and its feed-forward effect on osteolytic cancer metastases.

### Bisphosphonates improved muscle function in AI-treated OVX mice independent of muscle mass

**Figure 7:**
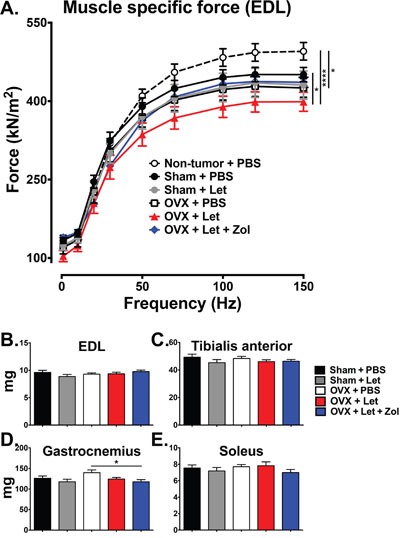
Ex vivo contractility of the EDL muscle **Panel A.** *Ex vivo* muscle specific force of the extensor digitorum longus (EDL) muscle was measured in tumor and non-tumor bearing mice. Data are expressed as mean force (ξ00B1;SEM) normalized to muscle size, and differences were determined by two-way ANOVA with Tukey's multiple comparisons test performed at 150Hz where *p<0.05 and ****p<0.0001. **Panels B-E.** Muscles of the hind limb were dissected and weighed, including the EDL, tibialis anterior, gastrocnemius, and soleus. Differences were determined by one-way ANOVA with Tukey's multiple comparisons test where *p<0.05.

Whole muscle contractility of the extensor digitorum longus (EDL) was measured at the termination of the study to evaluate the potential role of bone loss on muscle dysfunction in a state of AI-induced estrogen deprivation. Estrogen-replete mice with bone metastases (sham-PBS) had a reduction in muscle specific force of the EDL relative to non-tumor age-matched controls, and this reduction reached statistical significance when mice were ovariectomized (OVX-PBS) (Figure 7A). Addition of letrozole to OVX tumor-bearing mice (OVX-Let) led to a further reduction of muscle specific force production of the EDL, reaching statistical significance relative to both tumor and non-tumor and tumor-bearing sham-PBS mice (Figure 7A). This deleterious effect on muscle function in OVX-Let mice was partially reversed by the prevention of osteoclastic bone resorption through zoledronic acid treatment (OVX-Let-Zol) (Figure 7A). Observed changes in muscle specific force production were independent of changes in EDL muscle mass because force production measurements were corrected for size and weight of the EDL [29]. Furthermore, hind limb muscles including the EDL, tibialis anterior, gastrocnemius, and soleus collected at the termination of the study in cancer-bearing mice were similar between treatment groups (Figure 7B-7E), indicating that deficits in muscle specific force production in this study can likely be attributed to functional defects as opposed to muscle atrophy.

## DISCUSSION

The estrogen-replete and estrogen-deficient bone niches differ greatly as host environments for disseminated cancer cells due to the acute sensitivity of bone and marrow cells to changes in endocrine status. Estrogen acts directly on bone cells to regulate the lifespan of both osteoclasts and osteoblast, and inhibits T-cell production of inflammatory cytokines, which can further drive osteoclast activation and bone resorption [22, 30]. Increased bone resorption has been demonstrated in preclinical models, including OVX, to accelerate cancer progression in bone [30–32] presumably via release of matrix-derived growth factors, (e.g., TGFβ, IGF, FGF, PDGF), which stimulate tumor growth and expression of osteolytic factors that perpetuate a feed-forward cycle of bone destruction [20]. Using an ER-negative breast cancer cell line to avoid direct tumor growth inhibitory effects, our studies support the postulate that estrogen depletion by AI treatment alters the bone microenvironment in ways that can indirectly promote cancer cell homing, growth, and/or an osteolytic phenotype in bone. The assertion that AI-induced bone loss increased metastatic tumor growth was further supported by the finding that blockade of bone resorption by zoledronic acid reduced tumor burden in bone.

Direct anti-cancer effects of bisphosphonates have been pursued with relatively little evidence that physiologically relevant doses can directly elicit cancer cell apoptosis [33, 34]. Although direct anti-tumor effects of bisphosphonates have been shown *in vitro* [34], their anti-cancer activity *in vivo* continues to be attributed to indirect effects via inhibition of osteoclastic bone resorption [30]. Recent clinical reports have demonstrated differential anti-cancer effects of bone-targeted anti-resorptives in breast cancer patients depending on menopausal status. In the AZURE, ZO-FAST, and ABCSG-12 trials, zoledronic acid consistently improved disease-free survival in breast cancer patients, however, this effect was limited to 1) postmenopausal women and 2) premenopausal women undergoing AI therapy treatment (chemical menopause) [35–37], suggesting that the anti-tumor effects of zoledronic acid were reserved for estrogen deficient populations in a high bone turnover state. In light of our studies and the clinical link between bone loss and cancer progression, it will be important to consider the skeletal health not only of cancer patients undergoing AI therapy, but of patients undergoing any therapeutic intervention known to adversely affect skeletal health (e.g., GnRH agonists, glucocorticoids, radiation therapy).

We utilized the triple-negative MDA-MB-231 human breast cancer cell line to assess the impact of the microenvironment on tumor growth in the absence of effects on ER signaling. By contrast, adjuvant AI therapy in the clinical setting is prescribed to breast cancer patients with ER-positive primary tumors [8]. Clinical reports, however, indicate that in many cases ER status at the primary tumor site does not match the ER status of disseminated tumor cells [38], making our model relevant in the setting of advanced disease where metastatic tumor cell ER expression is lost. Use of an ER-positive breast cancer model (e.g., MCF-7) would be useful in determining the relative importance of the benefit of direct tumor growth suppression versus the risk of bone resorption-induced disease progression in the context of AI therapy. Breast cancer mortality results from tumor metastases rather than primary tumor growth [20], and bone is the preferred metastatic destination for breast cancer [39]. In the case of advanced metastatic disease, the growth inhibitory effects of AIs may be overridden by tumor-promoting signals from the bone microenvironment [40]. Thus, the prevention of bone loss in early stage breast cancer patients at the very onset of AI treatment could be of importance.

AI-induced arthralgia and muscle weakness are poorly characterized, yet are often so unmanageable that patients discontinue adjuvant AI therapy [10–14]. The identification of the molecular mechanism(s) underlying AI related muscle dysfunction could be critical in the development of interventions that can restore quality of life and improve drug compliance. In our studies zoledronic acid partially improved muscle function in AI treated mice with bone metastases, suggesting that osteoclastic bone resorption played a role in reduced skeletal muscle contractility in the setting of AI therapy. Recent work reported by our group identified bone-derived TGFβ as the mediator of muscle weakness in mice with bone metastases through the up-regulation of NADPH oxidase 4 production of reactive oxygen species leading to oxidation of the ryanodine receptor (RyR1) calcium channel complex in muscle resulting in SR Ca^2+^ leak and muscle weakness [25]. Release of bone-derived TGFβ during AI therapy-induced bone loss could have downstream effects on muscle contractility via a similar mechanism, a hypothesis currently under investigation in our laboratory. We speculate that AI-induced muscle weakness is likely multi-faceted in light of the finding that bisphosphonate treatment alone did not restore muscle function to the level of non-tumor control mice. It is possible that AIs elicit direct toxic effects on myocytes, a postulate that has yet to be tested. Selective estrogen receptor modulators (SERMs; e.g., tamoxifen), which interact with the ER and inhibit its activity in breast tissue while preserving bone have also associated with muscle weakness [15], suggesting that inhibition of ER signaling in muscle impacts muscle contractility in the absence of high bone turnover. Furthermore, muscle weakness associated with declining estrogen levels during menopause in healthy women can be rescued by hormone replacement therapy [41–43], providing further proof that ER signaling blockade contributes to muscle weakness in the AI-treated patient. Relevant non-tumor models and *in vitro* studies will be useful in determining the relative role of bone loss, ER signaling blockade, and potential direct drug toxicities on muscle function following AI and SERM therapy.

In summary, the causes of AI-induced musculoskeletal complications and the consequences of AI-induced bone loss are poorly characterized. Bone-derived factors released during an elevated state of osteoclastic bone resorption are known to have adverse effects on muscle function and can alter the bone microenvironment to favor breast cancer cell progression and perpetuate osteolysis. Our studies demonstrated that modulation of the bone microenvironment impacted tumor growth locally and muscle function systemically in AI treated mice. These findings emphasize the importance of considering the musculoskeletal health of cancer patients when selecting estrogen deprivation treatment options and the need to further investigate non-estrogenic therapeutic agents that can improve musculoskeletal outcomes in cancer patients.

## MATERIALS AND METHODS

### Animals

The Institutional Animal Care and Use Committee at Indiana University approved animal protocols for these studies in accordance with the National Institutes of Health Guide for the Care and Use of Laboratory Animals. Three-week female athymic nude mice were purchased from Harlan Laboratories (Indianapolis, IN) and housed in plastic cages with access to water and mouse chow *ad libitum* and maintained on a 12h light/dark schedule at 22 ξ00B1;2°C. After one week of acclimation, mice were anesthetized with a ketamine/xylazine cocktail and underwent bilateral ovariectomy (OVX) or a sham surgery under sterile conditions. Aromatase inhibitor (letrozole, 10ug/d), zoledronic acid (Zometa; 5μg/kg 3x/week), and vehicle (PBS, 50μl/d) treatments were administered daily via subcutaneous injection 24h after surgery and continued for the duration of the study. Blood was collected by retro-orbital puncture three days prior to surgery (baseline) and three weeks post surgery. Serum 17β-estradiol was measured by ELISA (Calbiotech) and expressed as percent of baseline.

### Bone microcomputed tomography (μCT)

Bone μCT was performed at the proximal metaphysis of the tibia using a high-resolution imaging system (μCT40; SCANCO Medical AG) on isoflurane-anesthetized mice. Bone μCT scans were acquired using a 17μm^3^ isotropic voxel size, 55kVp peak X-ray tube potential, 200ms integration time, and were subjected to Gaussian filtration. Trabecular bone microarchitecture was evaluated in the proximal metaphysis of the tibia in a region that began 0.4mm distal to the growth plate and extended distally 1.0mm. A threshold of 170mg HA/cm^3^ was used to segment bone from surrounding soft tissue. Trabecular bone outcomes included trabecular bone volume fraction (BV/TV; %), trabecular thickness (Tb.th; mm), trabecular number (Tb.N; mm^−1^), trabecular separation (Tb.Sp; mm), and connectivity density (Conn.D; mm^−3^). Scan acquisition and analyses were conducted in accordance with guidelines for use of μCT in rodents [44].

### Dual energy X-ray absorptiometry (DXA)

*In vivo* measurement of fat mass, lean mass and bone mineral density (BMD) was performed on anesthetized mice (ketamine/xylazine) using a PIXImus II densitometer (GE Lunar, Madison, WI) calibrated with a phantom of defined density. Body composition and BMD were measured at baseline on the day of surgery, at week six post-surgery, and at the termination of the study (week nine post-surgery), and data were expressed as percent change over time.

### Intra-cardiac inoculation procedure

Three weeks after OVX and sham surgeries, mice were inoculated in the left cardiac ventricle with MDA-MB-231 tumor cells, as previously described [27]. Briefly, tumor cell inoculation was performed percutaneously into the left cardiac ventricle of anesthetized mice in a supine position with a 26-gauge needle attached to a 1mL syringe containing 1×10^5^ cells suspended in 0.1mL sterile PBS. Visualization of bright red blood entering the hub of the needle in pulsatile fashion was indicative of correct needle placement into the left cardiac ventricle. Mice were followed for the development of osteolytic bone lesions by radiography on days 23 and 32 of the experiment. Osteolytic lesions were visualized in anesthetized mice in prone position using a digital X-ray imager (Kubtec) at 2.7x magnification. Lytic lesion area, reported as total lesion area (mm^2^) per animal in hind limb and forelimb long bones, was analyzed in a blinded fashion using ImageJ 1.48r software (National Institutes of Health).

### Histology

Hind limbs were removed from mice at the time of experimental termination, fixed in 10% neutral-buffered formalin for 48h and stored in 70% ethanol. Tibiae, femora, and humeri were decalcified in 10% EDTA for two weeks, processed using an automated tissue processor (Excelsior, Thermoelectric), and embedded in paraffin. Mid-sagittal 4.5μm sections were stained with hematoxylin and eosin (H&E) with orange G and phloxine to visualize new bone, and with tartrate-resistant acid phosphatase (TRAP) to visualize osteoclasts. Total tumor area was measured in H&E-stained mid-sagittal sections of the tibiae, femora, and humeri at 10x magnification without knowledge of experimental groups. Osteoclast cells were quantified in the mid-sagittal sections of tibiae in tumor-bearing hind limbs. Briefly, TRAP-positive multinucleated cells were quantified at 40x magnification along the perimeter of the tumor where the cancer cells interfaced directly with bone surfaces. Data were expressed as number of osteoclasts per mm of tumor-bone interface (OcN/mm), as previously described [27]. All sections were viewed on a Leica DM LB compound microscope outfitted with a Q-Imaging Micropublisher Cooled CCD color digital camera (Nuhsbaum Inc., McHenry, IL). Images were captured and analyzed using BioQuant Image Analysis Software version 13.2 (BIOQUANT Image Analysis Corporation, Nashville, TN).

### Measurement of muscle specific force

*Ex vivo* contractility of the extensor digitorum longus (EDL) muscles was determined as previously described [25]. Briefly, EDL muscles were dissected from hind limbs and stainless steel hooks were tied to the tendons of the muscles using 4-0 silk sutures and the muscles were mounted between a force transducer (Aurora Scientific) and an adjustable hook. The muscles were immersed in a stimulation chamber containing O_2_/CO_2_ (95/5%) bubbled Tyrode solution (121 mM NaCl, 5.0 mM KCl, 1.8 mM CaCl_2_, 0.5 mM MgCl_2_, 0.4 mM NaH_2_PO_4_, 24 mM NaHCO_3_, 0.1 mM EDTA, 5.5 mM glucose). The muscle was stimulated to contract using a supramaximal stimulus between two platinum electrodes. Data were collected via Dynamic Muscle Control/Data Acquisition (DMC) and Dynamic Muscle Control Data Analysis (DMA) programs (Aurora Scientific). The force–frequency relationships were determined by triggering contraction using incremental stimulation frequencies (0.5ms pulses at 1–150 Hz for 350ms at supra-maximal voltage). Between stimulations the muscle was allowed to rest for 3 min. At the end of the force measurement, the length and weight of the muscle was measured. To quantify the specific force, the absolute force was normalized to the muscle size calculated as the muscle weight divided by the length using a muscle density constant of 1.056 kg/m^-3^ [29]. The investigators were blinded to treatment of subjects.

### Statistical analyses

Differences were determined by one-way or two-way ANOVA, as appropriate, with Tukey's multiple comparisons test (GraphPad, Prism 6.0f). Results are expressed as mean ξ00B1;SEM and *p*<0.05 was considered significant.
